# 

**Published:** 1799-12

**Authors:** 


NEW PUBLICATIONS IN GERMANY.

*Vorſchläge zur Verbeſſerung der Hoſpitäler, &c.* Suggeſtions for the Improvement of Hoſpitals and other charitable Inſtitutions. By WILLIAM BLIZARD, F. R. S. and F. A. S. Tranſlated with additions, reſpecting the Hoſpitals and Medical Schools of London, Edinburgh, Bath, and Vienna. By Dr. I. A. ALBERS, phyſician at Bremen, 8vo. 128 pp. 1799, *Jena.* In the Academical Shop.

N. B. *We propoſe to give ample extracts from this highly intereſting little work, in our future Numbers; as the German Editor is a gentleman of unqueſtionable veracity, and perſonally known in this country, where he reſided the greateſt part of the years* 1796 *and* 1797.

*Chemiſche Briefe an Frauenzimmer, &c.*—Chemical Letters addreſſed to ladies; in which the principal ſubjects of Chemiſtry are explained in a popular manner; their application to economy, the arts, and amuſing experiments are pointed out, and inſtructions given for arranging and eſtabliſhing a laboratory. Two volumes, 8vo. with plates (Price 4 rixd. 20 groſch. or about 18s. Brit. curr.) *Leipzig.* 1779. Meyer.

*Kurze Darſtellung, &c.*—A Conciſe View of the Chemical Inquiries into the different Gaſes: by Dr. A. N. SCHERER, &c. 8vo. (6 groſch, or 1s.) *Ibid.*

*Kleine Mineralogiſche Schriften.*—Conciſe Eſſays on ſubjects of Mineralogy: by J. C. W. VOIGT, Counſellor of Mines, 8vo. Part I. with a plate. (20 groſch. or about 3s. 2d.) *Ibid.*

*Unterricht fur Aeltern, &c.*—Inſtructions for Parents, reſpecting the Dietetic Treatment of Infants at the Breaſt: by J. F. ZUCKERT, fourth Edition, enlarged and edited by Dr. L. FORMEY. 8vo. (8 groſch. or 1s. 4d *Berlin.*) Mylius.

*Umriſs des Zuſtandes, &c.*—A Sketch of the State of Surgery among the Ancient Romans, eſpecially in the time of Celſus; compared with, and applied to, Modern Surgery: by J. C. JÆGER; with a preface by the Aulic Counſellor and Prof. GRUNER, 8vo. (20 groſch. or about 3s. 4d.) *Frankfurt.* Jæger.

TO

### TO CORRESPONDENTS.

*We acknowledge the receipt of communications from Dr. Stokes, Dr. Scrimshire, Messrs. Ladaill, Brown, Grose, O. W. B. Brande, Pulley, Whyte, Latham, J. M. W. L. F. A. Viator, R. B. M. Henrique Xavier Baeta.*

*We are concerned to receive so many communications signed only with initials, particularly as several of our friends have suggested the propriety of very narrow limits to such articles.*

*The hint given us by an obliging Correspondeut, respecting the arrangement of the Index, could not be attended to in the present Volume, as it arrived too late; but we shall certainly pay due regard to his suggestion in our next.*

END OF VOL. II.

Printed by William Thorne, Red Lyon Court, Fleet Street.

### INDEX.

AIKIN, Mr. on certain fenfations of the eye 227
Alibert and Dumeril, Cits. their experiments with the gaftric juice 468
———, on odours 469
Alius, on quackery 274
Alkali, mineral, method of feparating it from common falt 390
Ammonia, the alexipharmic powers of 182
Anafarca of the liver, fingular cafe of 170
Anderfon, Dr. his engraving of the human body 190
Animal impregnation, remarks on 152
Antimony, new tincture of, by Mr. Heber 75
Aqua ammoni acetata, cheap way of faturating it 290
Aquilaria ovata, defcription of the 293

Bache, Dr. on a fucceffful cafe of afthma 140
Bandelow, A. D. on the medicinal virtues of vegetable fubftances 466
Barlow, Mr. on frictions with opium 107
Batty, Dr. his lectures 299
Baynton, Mr. on a new method of treating ulcers, &c." 92
———, on ulcers of the legs 197
Beddoes, Dr. his reply to Dr. Crichton 308
———, his "Effay on Confumption" reviewed 393
Belladonna, on the ufe of the, in cafes of mania 72
———, recommended in dropfy 182
Bellot, Cit. on hydrophobia 468
———, on a cafe of afcites 469
Benon, Cit. on the corrofive fublimate in the venereal difeafe
Bergman, Prof. his teft for cotton 83
Berlingheri, Cit. on the ufe of the gaftric juice in difeafes of the ftomach 467
Bernftein, Mr. on the trituration of mercury 389
"Biographia Medica" account of that work 396
Blair, Mr. his remarks on Mr. Owen's fragment 352
Bougies, metallic, by Mr. Smyth 85
Bree, Dr. on the ufe of the digitalis in confumption 314, 430
Brongniart, Cit. on the diffection of apes 480
Brown, Mr. on the hydrocephalus internus 142
———, on the treatment of hydrocephalus 258
———, on hydrocephalus continued 327
———, on a cafe of concuffion of the brain 407
Brugnatelli, Cit. on the ufe of lime, in calculous affections 75
———, on the inflammation of ethers 77
———, on a new acid ib.
Brugnatelli, Cit. on artificial cold 77
———, his experiments with lime 79
———, on the term foxygen 185
———, on the preparation of a folid gold ib.
Burferius, on the practice of phyfic 334
Cachexia Africana, account of the 171
Cæfarean operation, unfuccefsful cafe of the 74
———, a further ftatement of the cafe of E. Thompfon 472
Calf, remarkable ftructure of a 305
Campbell, Mr. A. his Inquiry into medical knowledge. 85
Caoutchouc, on the folution of 83
Carradori, Dr. on nocturnal birds of prey 79
———, on the refpiration of frogs and fifh ib.
———, his defcription of an apparatus for impregnating liquids 80
——— on Pierre Smith's experiments on animals ib.
Chauffier, Cit. on the Ormfkirk remedy 468
Chifholm, Dr. on the Cachexia Africana 171
——— on the eudiometer 387
Chriftie, Mr. on hepatitis 4
——— on gun-fhot wounds 442
Clutterbuck, Mr. his Remarks on the opinions of Mr. Hunter 86
——— on a cafe of hydrocephalus 247
——— on the treatment of ditto 154
Colman, Mr. on an extra-uterine fœtus 262
Confbruch, Dr. on the ufe of fnails in fiftulous ulcers 75
Confumption, hiftorical facts relative to the cure of 70
Correfpondents, anfwers to 96, 200, 304, 400
Crichton, Dr. on a note in Dr. Beddoes's "Contributions" 203
Croft, Dr. on the vaccine inoculation 391
Cuvier, Cit. on the hearing faculty of whales 480
——— on a fingular anatomical fubject 480

Davidfon, Dr. his experiments with the eudiometer 386
Davis, Dr. on inoculations with cow-pox matter 105
Deaths, lift of, by the Bills of Mortality for three months 320
Denman, Dr. on an abfcefs after childbirth 17
——— on a cafe of dropfy of the ovarium 20
Dennifon and Squire, Drs. their Lectures 299

Defcemet, Cit. on the irritability of the forrel-thorn 80
Digitalis, on the ufe of, in confumption 175
Difeafes, monthly lifts of 14, 15, 16, 111, 112, 208, 292 318, 319, 408
Drake, Dr. on the digitalis in confumption 267
——— on the cure of confumption 417
Duma', Prof. on Dr. Reid's " Effay on confumption" 51
Dumeril, Chauffier, and Dumas, Cits. their plans of an anatomical nomenclature 479
Dyce, Dr. on an old remedy for the tooth-ach. 269

Editors of the " Annales de Chimie," their experiments with acids 81
Emetic tartar, experiments with 74
Encyclopedia Botanica of Britifh plants announced
Epilepfy, treated by the argentum nitratum 173
Errata in Vol. I. 96
——— in No. 9. 400
" Effay on longevity," review of 93
Eudiometer, experiments with the 386
Evans, Mr. on the cow-pox inoculation 310
Excoecaria agallocha, defcription of the 293
Extractum taxi, remarks on the 85

Ferro, Dr. on the treatment of nervous fever 466

Fordyce, Dr. his " Third Differtation on fevers" reviewed 393

Fofter, Mr. on a cafe of monftrofity 226

Fothergill, Dr. on the dolor faciei 465

Fournier, Cit. on the medicinal properties of oxygen 38

Fox-glove, fome remarkable effects of 118

Frank, Dr. L. on the treatment of nervous fever 466

Franks, Mr. on typhus 195

Fries, M. his effay on the Stœchiometry of Richter 298

Fuller, Dr. quotation from his " Medicina Gymnaftica 71

Gaertner, M. on the conftituent parts of urine, &c. 298
Garnett, Dr. on the Moffatt waters 354
Gibfon, Dr. on bilious difeafes 194
Girard, Cit. his " Tableaux comparatifs, &c." 94
Girdleftone, Dr. on a cafe of diabetes 87
Göttling, Prof. on the origin of carbon 388
——— on the precautions in melting the heavy fpar ib.
——— on the Bononian phofphorus ib.
——— his method of preparing the the muriatic acid ib.
——— on azotic gas and phofphorus 389
" Gren's elements of chemiftry," tranflation of, announced 299

Gren, Prof. on the combuftion of phofphorus 389

Halle, Cit. on an extraordinary fœtus 480

Hecker, Dr. account of his " Elements of phifiology," in German 398

Henry, Mr. on frictions with opium 102

Hildebrandt, M. his experiments on the folution of mercury in the nitric acid 387

Holt, the Rev. Mr. on the cow-pox 401

Horn, Mr. on leeches 199

Hofe, M. his herbarium vivum, &c. 193

Huggan, Dr. on venefection and opium 233

Hull, Dr. his " Britifh Flora" reviewed

Hulfenkamp, M. his Latin differtation on ether 81, 82

Hunter, his natural hiftory of the teeth 184

Hutchinfon, Mr. his experiments with emetic tartar 74

Huzard, Cit. his " Obfervations, &c. 94

Hydrophobia, obfervations on the 173

Juch, M. on the fmell of nitric acid from fugar 298

J. Y. on Mr. Brown's obfervations on " Zoonomia" 276

Kafteleyn, Cit. on the cryftallization of potafs, &c. 81
———. on the flowers of ammoniac, 82
Kinglake, Dr. on the nature and properties of vital power 127
——— his tranflation of Trommfdorff's art of writing prefcriptions 191
——— on a cafe of inflammation of the glutei mufcles 285
Knebel, Dr. J. G. account of his German work 396

Lamb, hermaphrodite, Mr. Thomas, on an 1

" Lectures on diet and regimen," review of 301, 393, 493

Lentin, M. on the fubftance of the falling ftar 298

Leonhardi, M. on phlogifton and oxygen 83

Lenz, Dr. his Mineralogical Pocket-book 193

Lettfom, Dr. his hiftory of the tea-tree 192

Leveillé and Larrey, Cits. on the ufe of the muriat of barytes 469

Leveillé, Cit. on an extraordinary fœtus 479

——— on the retina of the human eye 479

——— on a fubject with an imperforate anus ib.

——— on a fingular anatomical fubject 480

Lewin, Dr. on the medicinal effects of yeaft 374

Lombart, Cit. on chirurgical cafes 46, 154

——— on compreffes moiftened with cold water, in cafes of hernia 46

Lombart, Cit. on the distortion of the abdominal viscera, by whalebone stays 47

——— on an abscess of the liver 48

——— on an insect discharged from the nose 49

Lowe, Mr. on the use of musk and ammonia 342

Lowitz, Prof. his experiments with vegetable acids 81

Lynam, Mr. on the aqua ammoniæ acetata 239

Maclean, Dr. on the digitalis purpurea 177

Marcet, Dr. on a case of diabetes 209

Marshall and Weisenthal, Drs. on worms in poultry 204

Marsillac, Cit. on a new method of inoculation 468

Medical Society, analysis of their memoirs 146

"Memoirs of the Medical Society," account of 396

Metallic tractors, observations on 85

Mills, Mr. on a case of uterine hydatids 447

Miller, Dr. on febrile diseases 373

Mitchill, Dr. on the affinities of nitric fluids to the bodies, &c. 180

——— on the application of septic fluids to explain some diseases of the bones 183, 380, 464

——— on soda, the alkaline basis of animal gall, &c. 383, 470

Moffman, Dr. on the digitalis in consumption 35, 238

Mussin-Puschkin, M. his experiments with vegetable acids 81

Mussin, M. his experiments with sulphur and phosphorus 185

Nooth, Dr. on the treatment of dysenteries, &c. 181

"Observations on the diseased Urinary Bladder," review of 303

Ogden, Mr. on the Cæsarean operation 476

Osborn and Clarke, Drs. their Lectures 191

Owen, Mr. his translation of an historical fragment 272

Parkinson, Mr. his "Table of Symptoms" 92

——— his chemical memoranda 191

——— his new work on health announced

Pearson, Dr. on the variolæ vaccinæ 97, 213

——— Mr. on a discharge of water through the navel 330

Persoon, M. his "Dissertationes Academicæ," &c. 192

Potass, on the purification of, 391

Proctor, Mr. on the concentration of vinegar 108

Publications, lists of new medical, in England, Germany, and France, 95, 199, 200, 304, 399, 400, 499

Pulley, Mr. on Dr. Vaughan's case of venesection 265

Quack medicines, letter on the suppression of 150

———, method of preventing the circulation of 392

Rahn, J. H. his medical magazine reviewed, 499

Redfearn, Dr. on inoculation with the cow-pox 23

Richter, Dr. on a particular species of dropsy 182

Ring, Mr. his remarks on the cow-pox 25

———, on venesection 346

Rosenmuller and Tilesius, Drs. their "Description of Caverns" 492

Rougnon, Cit. his "Medicine preservative, &c." 94

Rowlands, Mr. on two successful cases of lithotomy 287

Rudolphi, Dr. his Swedish Annals of Medicine 394, 498

Rush, Dr. on the hydrophobia 174

———, on the good effects of depletion 178

———, on the nature and cure of the gout 376

Sachse, Dr. on a dropsy of the liver 170

Sandford, Mr. on a remarkable structure of a calf 305

Saumarez, Mr. on generation, &c. 242, 321

Scherer, Dr. on the extraction of sugar from the beet-root 188

———, on the chemical action of light 297

———, on an apparatus for bleaching ib.

———, on the muriat of barytes ib.

———, remarks on the production of sulphur ib.

Schrader, M. his Botanical Journal 193

Schwarz, M. on the decomposition of mercury 390

Seamen, Dr. his lectures on Midwifery 190

Sheldon, Mr. his method of preserving pathological subjects 189

Sherwen, Dr. on the digitalis in consumption 175

Silver, nitrat of, in epilepsy 70

Simmons, Mr. on Mr. White's treatment of sphacelus 12

———, on the fatal effects of the Cæsarean operation 231

———, on the same subject 437

Sims, Dr. on pure ammonia in pregnancy 205

———, on the Cæsarean operation 433

Snails, use of, in fistulous ulcers 75

Society, Medical, of London, analysis of the memoirs of, 29

Spasmodic asthma, remarks on the cure of 169

Sprengel, Prof. his "Historical View of Surgery," continued 54, 155, 278, 364, 453

——————, his History of Anatomical Discoveries 60, 160, 281, 368, 459

Spry, Mr. on a case of hydrocephalus internus 431

St. Fond, on the solution of caoutchouc 83

Struve, Dr. his translation of Bacon, on the prolongation of life 496

——————, essay on resuscitation 497

"Swedish Annals of Medicine," account of that work 399

"Tableau du regne Vegetale," &c. account of 397

Tassaert, Cit. his experiments on the muriatic acid 78

Thomas, Mr. H. L. on an hermaphrodite lamb 1

Tiebol, M. his experiments with mercury 82

"Treatise on Febrile Diseases," review of 300

Unzer, Dr. on the cure of spasmodic asthma 291

Urine, incontinence of, cured by hepatised ammonia 288

Vage, Dr. on inveterate ulcers 349

Van Mons, on the impregnation of water 80

——————, on fulminating substances 185

——————, on the black oxyds of mercury 186

——————, on the non-oxygenability of the sulphuric acid ib.

——————, his discovery of a white oxyd of mercury 188

Van Mons, on the beet-root 189

——————, his method of separating soda from sea salt 391

Vaughan, Dr. his answer to Mr. Pulley 405

Vision, J. Y. on distinct, at different distances 331

Wainewright, Mr. on an uncommon tumour of the abdomen 362

Walker, Mr. his "Memoirs of Medicine" 94

Walters, Mr. on the dysentric effects of nitrous acid 179

Ward, Mr. on the effects of opium 6

——————, his cases of inoculation for the cow-pox 134

Welter, Cit. his experiments with the nitric acid 76

Wendland, Mr. his "Description of Heaths" 492

Whately, Mr. "On the cure of wounds and ulcers of the legs" 88

——————, on the same subject 195

White, Dr. on a case of epilepsy 173

Whyte, Dr. on the prevention and cure of dysentery 283

Wildenow, Prof. on the aromatic wood of aloe, &c. 293

Williamson, Dr. on the ill effects of blood-letting in putrid fevers 179

Wilkinson, Mr. on the powers of electricity, 9

——————, on the air bladder of fish 360

Willich, Dr. review of his "Lectures" 301, 393, 493

Wurzer, M. on the use of the nitric acid 82

——————, on Hahneman's soluble mercury ib.

Yeats, Dr. his answer to Dr. Lubbock 98

Zanthoxylon, on the efficacy of 3[illegible]

### *References to the Plates.*

*Page*

I. A Plate, representing the Organs of Generation of an Hermaphrodite Lamb, two months old; communicated by Mr. H. Leigh Thomas: (No. VI.) described - 1—4.

II. A coloured Plate, exhibiting the Excœcaria Agallocha and Aquilaria ovata; by Prof. Wildenow, of Berlin; (No. VIII.) described - - - - - 293—296.

III. A Plate, representing a remarkable Structure and Appearance of the External Organs of Generation in a Calf: communicated by Mr. W. Sandford; (No. IX.) described - - - - - - 305—308

